# Pelvic hemangiopericytoma: the role of diffusion weighted imaging in targeting the biopsy site and in monitoring the tumour response to radiotherapy

**DOI:** 10.2478/v10019-010-0045-5

**Published:** 2010-10-14

**Authors:** Evangelos Perdikakis, Eelco de Bree, Elpida Giannikaki, Evangelia G. Chryssou, Christine Valatsou, Apostolos Karantanas

**Affiliations:** 1 Department of Radiology, University of Crete, Stavrakia, Heraklion, Crete, Greece; 2 Department of Oncological Surgery, University of Crete, Stavrakia, Heraklion, Crete, Greece; 3 Crete University, Department of Pathology, Stavrakia, Heraklion, Crete, Greece

**Keywords:** CT, MR imaging, diffusion weighted imaging, biopsy, hemangiopericytoma, pelvis, radiotherapy

## Abstract

**Background:**

Despite advances in imaging, the accurate characterization of soft tissue tumours remains a challenging task. Furthermore, the interpretation of post treatment changes and evaluation of tumour response to therapy is another complicating issue regarding soft tissue tumour imaging.

**Case report.:**

Herein, a patient with a pelvic hemangiopericytoma, by whom different diagnostic imaging methods were used, is presented.

**Conclusions:**

Diffusion weighted imaging (DWI) might provid useful information in guiding biopsy and enabled monitoring of the radiation therapy results.

## Introduction

Soft-tissue tumours often require a combined therapeutical approach including surgical resection, chemotherapy and radiation therapy.^1^ In selected inoperable cases, the radiation therapy and chemotherapy may be the only treatment options.^2^ Although MR imaging is considered the modality of choice for the diagnosis and the follow-up of the applied therapeutic scheme, diagnostic dilemmas may rise both for the clinician and the radiologist.

The accurate assessment of a positive or negative tumour response to radiation or to chemotherapy plays an important role in the appropriate patient management.^3^ Distinguishing between the normal and anticipated therapeutic results from tumour recurrence is another difficult issue in post treatment imaging.^3^ The application of diffusion weighted imaging (DWI) in oncological imaging has gained enough attention and enthusiasm during the past decade with increasing efforts of its extracerebral applications.^4^,^5^ DWI might help in the differentiation of various benign and malignant alterations.^5^,^6^ The basic principle and the main advantage of this rather new method is the ability to study the water movement at a cellular level.^4^ Impendance in the random water motion is expressed as the diffusion restriction and the qualitative and the quantitative analysis is now feasible due to technological improvements and newer MR techniques.

We describe the radiologic findings of a pelvic hemangiopericytoma that was treated solely with the radiation therapy. Hemangiopericytoma is a rare soft tissue tumour that usually breaks out during adulthood.^7^ It is considered a malignant tumour of vascular origin that commonly requires a combined therapeutical scheme.^7^ In the present case report the emphasis is given on the application of DWI both prior to the diagnosis as well as in the imaging follow-up.

## Case report

A 58-year-old female patient presented to the surgical oncology department with a 6-month history of abdominal discomfort, constipation and pain in her back irradiating to the right femoral and inguinal area. An increased frequency of micturition initiated at the same time period, was also reported. The laboratory examinations were within normal limits indicating only a minor sideropenic anaemia and hyperlipidemia. The physical examination of the abdomen was negative but the digital vaginal examination showed the presence of a palpable mass in the right pelvic wall. A subsequent US examination, performed elsewhere (not available), verified the presence of the lesion and characterized it as highly suspicious for malignancy.

**FIGURE 1.**
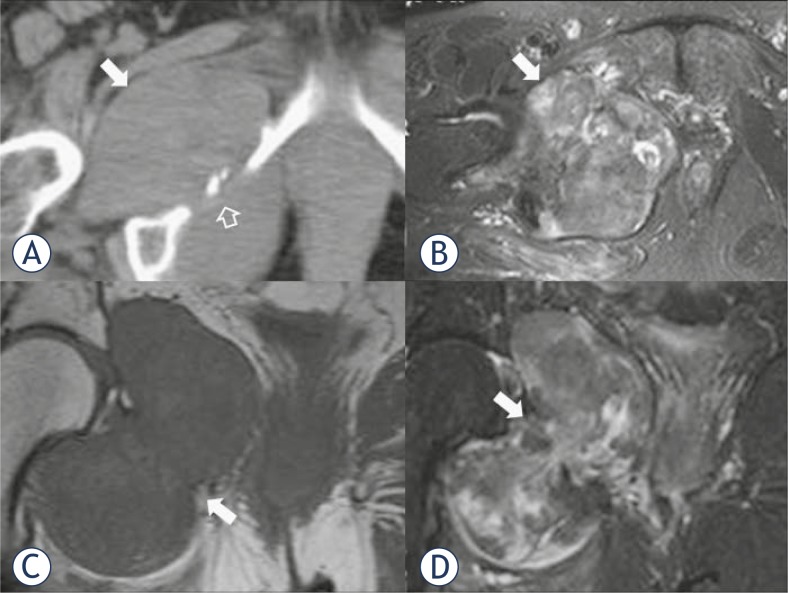
The axial (A) noncontrast multidetector computed tomography (MDCT) image shows a large isodense mass occupying the right ischiorectal fossa and extending in the femoral anatomic compartment posteriorly to the pectineus muscle (solid arrow in A). Note invasion and destruction of ischiopubic ramus (open arrow in A). The axial (B) fat suppressed T2-w MR image demonstrates a heterogeneous, with predominantly high signal intensity mass (arrow in B). The coronal (C) T1-w and the coronal (D) short tau inversion recovery (STIR) images demonstrate a large bicompartmental mass (with pelvic and femoral component) that shows an “hour-glass” configuration (arrows in C, D).

A following multidetector computed tomography (MDCT) examination demonstrated that the lesion was located in the right ischiorectal *fossa*, invaded the obturator muscles and the right ischiopubic *ramus* and extended in the femoral anatomical compartment displacing anteriorly the right pectineus muscle (Figure 1A). The tumour was isodense to skeletal muscles in the non contrast series and showed intense enhancement in the arterial and delayed phase.

**FIGURE 2.**
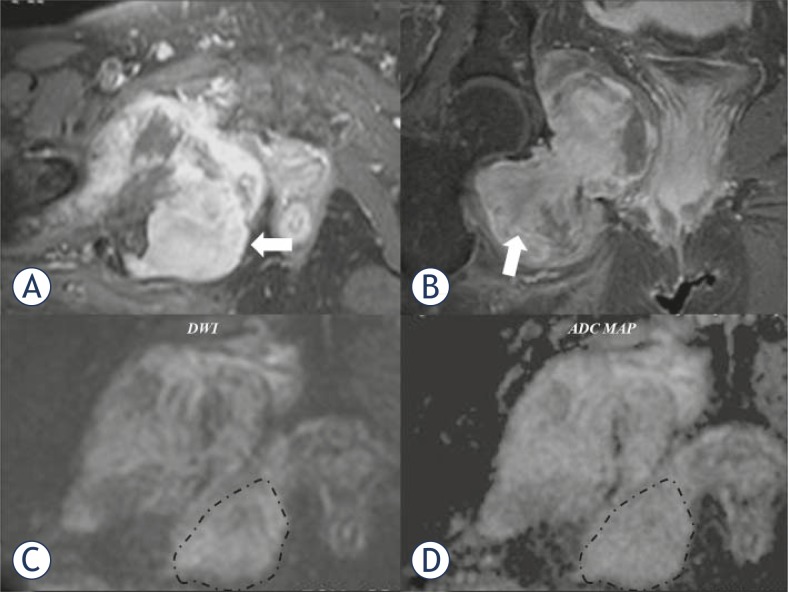
The axial (A) and coronal (B) contrast enhanced fat suppressed T1-w MR images show intense enhancement of the lesion (arrows). The diffusion weighted imaging (DWI) (C) and the corresponding apparent diffusion coefficient (ADC) map (D) demonstrate signs of increased restriction in diffusion, a finding in keeping with the high cellular nature of the tumour. Area in dashed line corresponds to the biopsy selected site.

The patient was referred for additional MR imaging evaluation. MR imaging showed the tumour to exhibit low signal intensity on T1-w and high but heterogeneous signal intensity on T2-w images (Figures 1B–D). Intense and heterogenous enhancement was shown following the gadolinium injection (Figures 2A–B). The application of DWI showed areas of increased signal intensity that were indicative of a high cellular tumour (Figures 2C–D).

**FIGURE 3.**
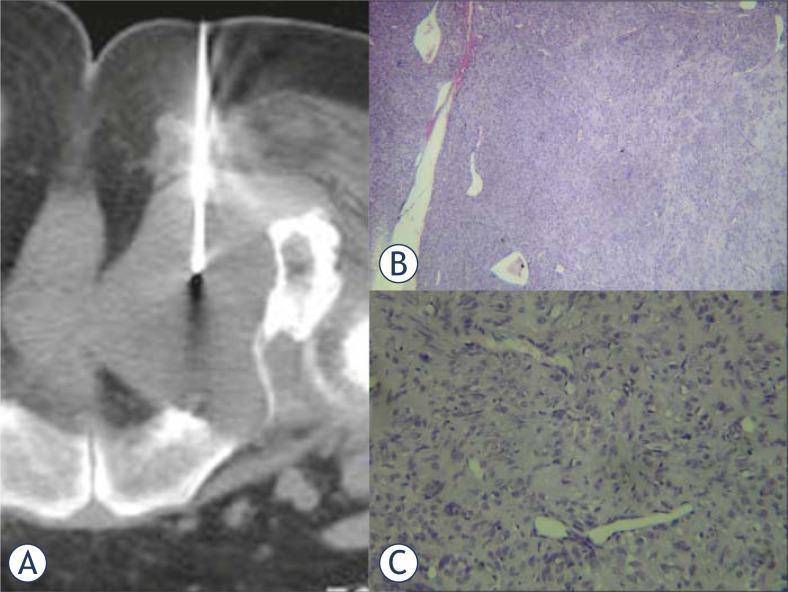
(A) CT guided biopsy of the lesion with an 18G true cut needle. (B) Power view (H&E stain, X100) of the biopsy, showing a mesenchymal tumour with a hemangiopericytoma-like pattern, composed of spindle cells arranged in short bundles and the characteristic vascular pattern with a partial “staghorn” configuration. (C) High power view of the spindle cell areas (H&A, X400). The spindle cells show no atypia, mitosis or necrosis.

The patient underwent a CT-guided biopsy targeted in the intratumoural areas that corresponded to areas of the maximum diffusion restriction and the diagnosis of hemangiopericytoma was established (Figure 3). Findings, consistent with malignant biological behaviour, such as necrosis, cellular *atypia* and high mitosis count, were absent. MDCT examinations of the chest and abdomen were negative for metastatic disease.

Due to the local extent and infiltration the tumour was considered unresectable. The radical excision of the tumour should have led to hemipelvectomy, which was not justified in absence of apparent malignant features. In a multi-disciplinary meeting the initial treatment with radiotherapy was suggested. Radiotherapy consisted of 50.4 Gy (28 × 1.8 Gy, 3 fields) external beam radiation with weekly intravenous administration of 20 mg Caeylix and 20 mg Cisplatin as radiation sensitizers. Unfortunately, radiotherapy was applied as a palliative radiation and not as the primary treatment, because the patient did not give her full consent in fear of the possible radiation injuries and complications. Four weeks after finishing radiotherapy the patient revisited the surgical department, with new imaging studies. The MDCT examination of the chest and abdomen was again negative for metastatic disease, while the MR imaging showed a small reduction in tumour size. Gradually, there was a complete relief of symptoms. The radiotherapist considered that there was no indication to persuade the patient to complete radiotherapy to the full therapeutic dose especially after such a long period without the treatment and preferred the remaining dose to be administered in case of the tumour growth.

**FIGURE 4.**
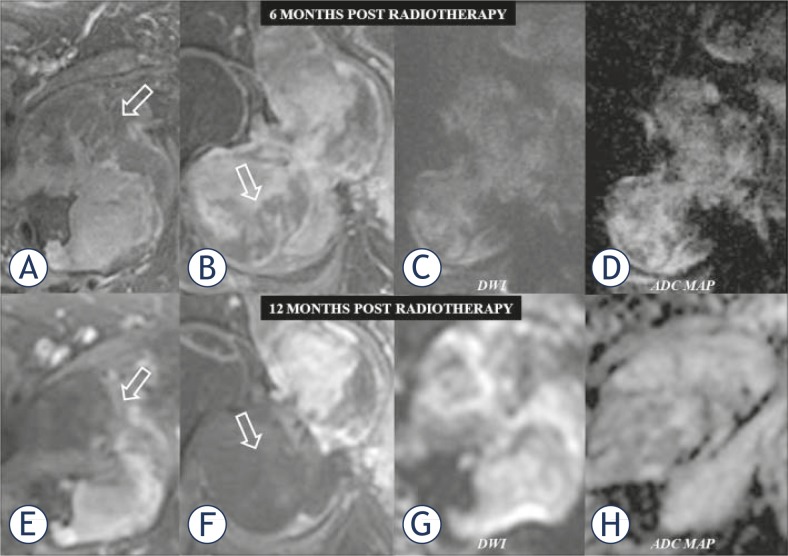
Assessment of the tumour response 6 months (upper series) and 12 months (lower series), following radiotherapy. Fat suppressed post gadolinium T1-w MR images in the axial (A, E) and coronal plane (B, F), diffusion weighted imaging (DWI) in the coronal (C) and axial (G) plane and the corresponding apparent diffusion coefficient (ADC) maps (D, H). Note consecutive toumoral necrosis (no enhancement), which is more pronounced in the femoral compartment of the mass (open arrows). The increased signal in the femoral part of the lesion (C, G) as well as in the ADC map (D, H), represents tumour liquefaction and thus an evidence of positive response to radiotherapy. For comparison purposes the chosen DWI performed 6 and 12 months following treatment, are of the same *b* values.

Six months following treatment the patient was scheduled for a follow-up MR imaging, which verified a decrease in the lesion’s enhancement characteristics post gadolinium. Intratumoural areas that did not enhance were considered to be a positive response to therapy (Figures 4A–D). Furthermore, DWI showed a slight increase in the intralesional signal intensity of the femoral component of the mass with a concomitant signal increase in the apparent diffusion coefficient (ADC) map. This finding was considered an indication of the reduction in the restriction of water molecules and correlated with the nonenhancing areas (Figures 4A–D). In other words, the mass seemed to begin to become less cellular and liquefied as a result of the radiation therapy.

The effectiveness of the therapy was further assessed and established on a follow-up MR imaging study twelve months post radiotherapy. Despite no change in the outer shape and contour of the tumour, more than 60% of its volume showed no evidence of the enhancement and at the same time DWI showed further indications of the unimpaired diffusion as a result of possible tumour necrosis and liquefaction (Figures 4E–H). One year after radiotherapy the MDCT examination of chest and abdomen remained also negative for eventual metastatic disease and the patient was free of symptoms.

## Discussion

Hemangiopericytomas are rare soft tissue tumours that usually occur in adulthood during the 4^th^ and 5^th^ decade.^7^ Various locations of the tumour have been reported with an increased prevalence in the lower extremities and retroperitoneum.^7^,^8^ Histopathologically the tumour arises from the Zimmerman pericytes around either capillaries and/or postcapillary venules and thus on the microscopic examination the characteristic finding is the identification of capillary channels surrounded by spindle-shaped cells.^8^,^9^ Clinically, a usually painless mass located in the deep soft tissues may present with mass effect on adjacent organs and structures.^7^–^9^ The surgical resection is the treatment of choice but the tumour tends to recur even after the total macroscopic resection. Local invasion or relapsing tumours may preclude the surgical treatment. Distant metastases occur in the lungs or bones and are observed in high-grade, poorly differentiated or undifferentiated tumours (grade III–IV).^10^ In inoperable cases or as an adjuvant treatment, radiation therapy can be applied.^11^

In our case a bicompartmental tumour was identified (pelvic and femoral location) that initially presented due to the local mass effect and pressure on adjacent anatomical structures. The correct tumour characterization was feasible by means of CT-guided biopsy and the targeting of the biopsy site was done by using the information from the DWI sequences.

The physical basis of diffusion-weighted imaging relies on the random motion of the water molecules (Brownian motion) that was first described by Einstein in 1905.^12^ The restriction of this random movement of water molecules that is present within biologic tissues (due to impendance caused by cell membranes and intracellular organelles) as well as differences between intra-extracellular spaces is the basis of DWI.^13^–^14^ As a consequence, increased tissue cellularity that is present in highly cellular neoplasms may impede the free movement of water molecules and may reflect as the increased restriction in DWI images. On the contrary, the restoration of a less tight cellular environment (post therapy) may be imaged as an increase in the molecular diffusion.^15^–^17^

A quantitative analysis can be achieved with the acquisition of ADC maps from diffusion images obtained at different b values. Consequently, we used DWI in two modes. Firstly, the selection of the target site for biopsy was based on the area that demonstrated the maximal restriction in diffusion. This was chosen by correlating DWI and ADC maps. The interpretation of diffusion weighted images allows the detection of focal solid and cystic lesions.^15^–^17^ The more cellular areas can provide better diagnostic results following CT-guided biopsies obviating thus the need for repeating an interventional procedure. This application may be of extreme value in cases of soft tissue tumours that contain areas of intratumoural necrosis. A biopsy targeted at the necrotic area would turn out to be non diagnostic and repetition would be needed, increasing thus the cost and possible complications.

Secondly, the tumour’s response to radiotherapy was also assessed with DWI. We correlated the post gadolinium MR images with DWI and ADC images, both before and after the treatment. This correlation showed that non enhancing areas demonstrated the diffusion restoration on a one-year follow-up studies, a finding that was considered a positive response to the treatment. The observation of tumour necrosis, which is thought to be representative of a positive therapeutic response, is of paramount clinical importance. Hamstra *et al.* and Dudeck *et al.* have recently studied this novel application of DWI in oncology and initial results are considered encouraging in evaluating the anticancer treatment.^15^,^17^ This role of DWI in monitoring the tumour response to radiotherapy or chemotherapy might be of extreme value in the future. Patients with contrast media allergy and inability to be studied with gadolinium infusion could have an adjuvant MR tool for the assessment of the therapeutic scheme.

In conclusion, our case suggests that DWI may be used for targeting the appropriate biopsy site and for the assessment of the radiation treatment. However, a larger series of patients is needed to confirm this application and establish a feasible and reproducible diffusion weighted MR imaging protocol for the follow-up of the oncological patient.
